# Metastatic carcinoma of the oral region: An analysis of 21 cases

**DOI:** 10.4317/medoral.21566

**Published:** 2017-04-08

**Authors:** Yeon-Hee Lee, Jae-Il Lee

**Affiliations:** 1 DDS, PhD. Department of Orofacial Pain and Oral Medicine, Kyung Hee University Dental Hospital, Seoul, Republic of Korea; 2 DDS, PhD. Department of Oral Pathology, School of Dentistry, Seoul National University, Seoul, Republic of Korea

## Abstract

**Background:**

Metastatic carcinoma to the jaws and oral region are very rare, representing less than 1% of all oral tumors. Unfortunately, oral metastasis is usually manifestation of an advanced stage of primary cancer, and indicates widespread disease and poor prognosis.

**Material and Methods:**

In this retrospective study, a total of 2039 patients with history of oral malignant tumor between 1980 and 2012 at Seoul National University Dental Hospital were evaluated. We analyzed the dental and medical records, and histopathological database of 2039 patients to assess the prevalence of oral metastasis of carcinoma in terms of sex and age, as well as, the most common origin of primary cancer, and prevalent site and histopathological type of metastatic carcinoma.

**Results:**

Among 2039 patients, 21 (1.03%) were finally diagnosed with metastatic carcinoma of the jaws and oral region. Among the 21 patients, only 11 had a working diagnosis as oral metastasis upon clinical evaluation before performing a biopsy. The mean age at the time of diagnosis with a metastatic carcinoma was 56.86, and there was a male preponderance. Metastatic carcinoma was more frequent in the jaws than in the soft tissue, especially in the mandible compared to the maxilla. The most frequent primary site was the lungs, followed by the liver and breasts. The predominant histopathological types were hepatocellular carcinoma and adenocarcinoma. Patient outcomes indicated a poor prognosis with the time from the appearance of the metastasis to death was only 12 months.

**Conclusions:**

According to these cases, oral metastases of carcinoma were exceedingly rare in Koreans. It can allow the clinicians take into account the possible presence of metastases and lead to early diagnosis.

** Key words:**Metastasis, jaws, oral region, mandible, Korean.

## Introduction

Approximately 5% of all malignant tumors involve the oral region. Among them, metastatic tumors of the jaws and oral cavity are very rare, accounting for approximately 1% of all oral tumors (1,2). Hence, diagnosing a metastatic tumor in the jaws and oral cavity is very challenging for the both clinician and pathologist because of its rarity. Understanding metastasis is of great importance for the clinical management of cancer because min most cases, cancer-related mortality is associated with disseminated metastatic lesions. In addition, in most cases, cancer patients with widespread and disseminated tumors have significantly poorer prognoses than those with localized tumors (3). Unfortunately, several steps of carcinogenesis have already been explored (4,5), and the complex pathogenic mechanisms for spreading of primary tumor cells to the oral lesion are still uncertain (6). Although the metastatic site and the primary tumors might not always be directly connected through the blood vessels, lymphatic vessels, or serosal surfaces (7), distinct and various sites might be involved in the metastatic process.

Metastatic carcinoma, the major type of metastatic cancer, can affect both the osseous and soft tissues. The most common site for bone metastasis in the head and neck region are the jaws, comprising more than 70% of all malignant metastatic tumors in the oral cavity (2). Many researchers have reported that metastasis to the oral soft tissue is rare compared to that to the jaws (2,8,9). In the soft tissue, the most frequently involved site is the attached gingiva, followed by the tongue. The tongue has a rich vascular supply and can easily be involved in cancer metastasis (10). According to Bernstein *et al.*, cancer cells can also be transfer to the nasal cavity and paranasal sinuses (11).

Hashimoto *et al.* reported that metastatic carcinoma of the mandible was confirmed at autopsy in 16% of the patients with malignant tumors (12). Fifty percent of all oral metastases occurs from the primary carcinoma of breast, lung, and kidney. The remaining are from the prostate, thyroid, gastrointestinal tract, suprarenal, uterus, and bones (13). There are sex differences in the primary sites of metastases. In women, the most common sources of tumors metastatic to the oral region are primary cancers of the breasts and the reproductive organs, followed by the thyroid gland. In men, they are usually metastasized from the lungs and kidneys (14).

Despite the recent advances in diagnosis and treatment, prognosis still mostly depends on the stage of cancer at the diagnosis of the oral cavity lesion. If a metastasis can be quickly identify, while it is still localized with a lower stage or grade, the prognosis is better (15). However, oral metastases usually indicate widespread disease and poor prognosis. The time elapsed from the appearance of the metastasis to death is usually only a few months (2).

We aimed to evaluate the prevalence of metastatic carcinoma in the jaws and oral region in Korean patients indirectly, at a single institution, through direct analysis of our data over a 32-year period. We analyzed its clinical characteristics and histopathological features in order to improve our knowledge of the nature of oral metastatic carcinoma, which can allow the clinicians and oral pathologists take into account the possible presence of metastases.

## Material and Methods

All patients who were diagnosed with oral malignant tumors between January 1980 and December 2012 at the Department of Oral Pathology of Seoul National University Dental Hospital were enrolled in the present study. We retrospectively analyzed the histopathological, dental, and medical records of 2039 patients with oral cancer to assess the prevalence of metastatic carcinoma to the oral region. Cases satisfying the diagnostic criteria for oral metastatic carcinoma as recommended by Stern and Shepard (9) were included. All patients definitively diagnosed with metastatic carcinoma of the jaws and oral region based on complete information regarding the primary site, clinical presentation, radiologic findings, and histological features. Thus, we evaluated and analyzed the diagnosis of the primary lesion, the site of the primary tumor, time to metastasis, the choice of treatment (chemotherapy and/or radiation therapy), recurrence, and/or mortality, as well as the patient sex and age. The diagnosis for each patient was reconfirmed through microscopic examination of the cancer biopsy samples. The study was conducted in accordance with the ethical principles of the Declaration of Helsinki, and the protocol was approved by the institutional review board of Seoul National University Dental Hospital.

## Results

- Prevalence

Over the last 32 years at our hospital, 2039 cases of malignant tumors in the jaws and oral region, including carcinomas (1981 cases), sarcomas (47 cases), melanomas (5 cases), and other unspecified malignant neoplasms (6 cases) were diagnosed. Of the 2039 malignant cases, 21 patients had metastatic carcinomas. The proportion of metastatic carcinomas accounted for 1.03% of all oral malignant tumors, and 1.06% of all oral carcinomas.

- Age and sex distributions

Most patients who were diagnosed with tumors metastatic to the oral region (18/21 cases, 85.7%) were in their fourth to seventh decades. The mean age at the time of diagnosis with a metastatic carcinoma was 56.9 (range, 31-83) years. The mean ages of the men and women were 60.2 and 52.8 years, respectively. The mean age of patients with jawbone metastases (56.2 years) was lower than that of patients with oral soft tissue metastases (59.0 years) and the mean age of patients with metastases to the mandible (59.4 years) was slightly lower than that of patients with metastases to the maxilla (60.0 years). Metastatic carcinomas were more prevalent in men (13/21 cases) than in women (8/21 cases), and the men to women ratio was 1.63:1. The patients with oral metastatic carcinomas were predominantly men, whereas, the patients with oral soft tissue metastases were predominantly women, with a men to women ratio of 1:2 ( Table 1, Table 2).

**Table 1:**
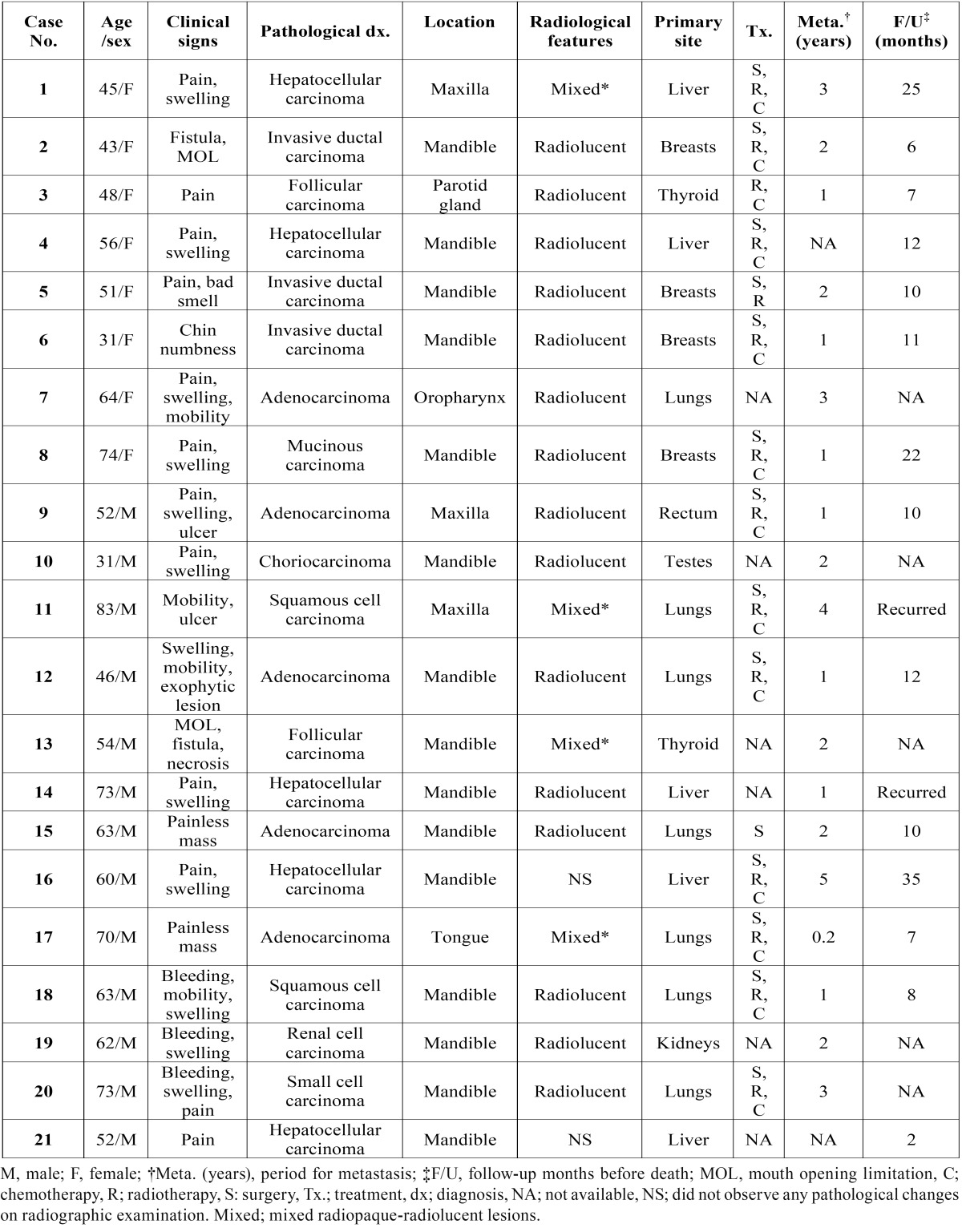
Clinical and radiological features of patients with metastatic carcinoma.

**Table 2:**
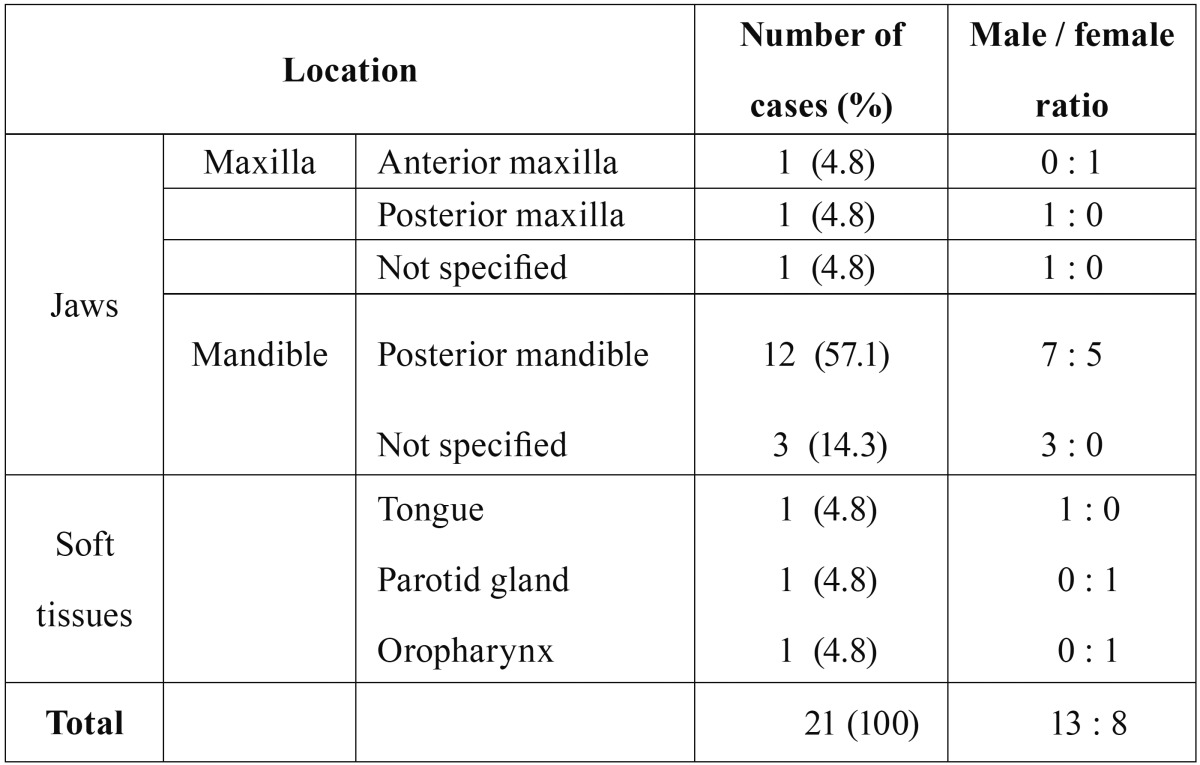
Sites of metastases in the jaws and oral region.

- Primary site

Several primary malignancies have the potential to metastasize to the oral region. In the present study, oral metastases most frequently originated from the lungs, liver, breasts, thyroid, and 1 case each from the kidneys, bladder, and the testes were observed. The site of origin differed between the sexes; for men, the most common site was the lungs, followed by the liver, thyroid, rectum, kidneys, and the testes. For women, it was the breasts, followed by the liver, lungs, and the thyroid ( Table 1, Table 3).

**Table 3:**
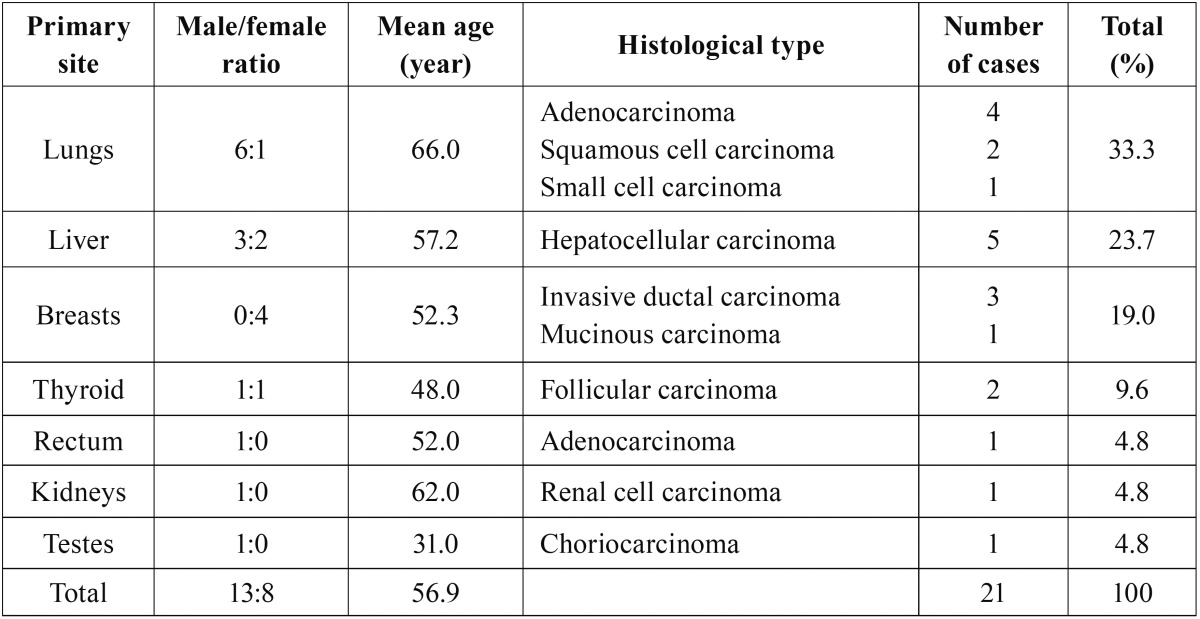
Histological type according to the primary site.

- Metastatic carcinoma of the oral region

Primary tumors metastasized to the jaws (18/21 cases) in the oral region more frequently than they did to the oral soft tissues (3/21 cases). In the jaws, the mandible was the most common location (15/18 cases; 83.3%), followed by the maxilla (3/18 cases; 16.7%). In the soft tissues (3/21 cases), the tongue (1/3 cases), parotid gland (1/3 cases), and the oropharynx (1/3 cases) were the sites of metastases. In the mandible, the molar area was the most commonly affected region (8/15 cases, 53.3%). There were 3 cases (20%) of non-localized spread throughout the entire mandible, followed by 1 case (6.7%) each involving the ascending ramus, mandibular angle, mandibular condyle, and the retromolar trigone. In the maxilla, there were 2 cases involving the molar region (66.7%) and 1 case of spread throughout the entire maxilla (33.3%). There was 1 case (33.3%) involving the maxillary incisor region, which had not been previously reported ( Table 2).

- Clinical presentation and radiological findings

The mean time elapsed from the appearance of oral symptoms to diagnosis was 3 months. The clinical presentations of the oral metastatic lesions differed between various sites. More than half of the patients (57.1%) complained of pain and swelling at the area of metastasis. During oral examination, we noted common clinical symptoms with increased dental mobility in 4 (19.0%), bleeding in 3 (14.3%), limitation of mouth opening in 2 (9.5%), and chin numbness in 1 (4.8%) out of the 21 cases of oral metastasis (Table 1). Radiographic examination revealed a radiolucent lesion in 15 (71.4%), and mixed radiopaque-radiolucent lesions in 4 (19.0%) out of the 21 cases. However, in 2 out of 21 cases (9.5%), the radiographs did not show any pathological changes.

- Histopathological findings

The histopathological diagnoses of the oral metastatic carcinomas are listed in  Table 1 and  Table 3. Metastasis from the lungs was observed in 7 out of 21 cases, and the most common type was lung adenocarcinoma (4/7 cases), followed by squamous cell carcinoma (2/7 cases), and small cell carcinoma (1/7 cases). Metastasis from the liver was observed in 5 cases, and the histological type for all the cases was hepatocellular carcinoma, which is the most common type of liver cancer. The patients with metastases from the lungs had a mean age of 66 years and they were the oldest compared to the patients with metastases from other sites. Patients with metastases from the testes were the youngest with a mean age of 31 years. The patients with primary follicular thyroid carcinoma, had a mean age of 48.0 years, which was 10 years younger than the mean age of the 21 patients (56.9 years) with oral metastases.

- Outcomes

The average time elapsed from the diagnosis of the primary tumor to the appearance of the jaw and oral region metastases, was 1.9 years (range, 1 month to 5 years). Various treatment methods including lobectomy, hepatectomy, radical mastectomy with radiotherapy, and chemotherapy were used in the treatment of the primary cancers. However, the patient outcomes were very poor. The average time to death after the diagnosis of a metastatic carcinoma in the oral region was 12 (range, 2-35) months. Ten patients (47.6%) died within 1 year of diagnosis, and the >2-year survival rate was 5%. Three patients (14.3%) showed metastases to organs and tissues other than the oral region; 1 case involved breast cancer metastasis to the lungs and scapula, 1 case involved primary lung cancer metastasized to the small intestine, and the last case involved primary lung cancer metastasized to the brain.

## Discussion

Metastatic carcinoma to the jaws and oral region are rare and account for approximately 1% of all malignant oral tumors (2). In the present study, among the 2039 patients who were diagnosed with oral malignant tumors, only 21 patients had oral carcinomas metastasized from other organs during the last 32 years. Most patients (85.7%) were in their fourth to seventh decades, with a mean age of 56.9 years. This finding is consistent with the results of previous studies, which reported that oral metastatic tumors occur in patients aged 40-70 years (16,17). The patients with jawbone metastases were younger than the patients who had oral soft tissue metastases, which is consistent with the findings of a previous study (18).

Whether oral metastasis has any predilection toward a particular sex is still controversial. The men to women ratio for the 21 patients with oral metastases, was 1.63:1, whereas, oral soft tissue metastases showed a predominance for women. Hirshberg *et al.* reported that metastases to the jaws have a female predilection (18). Schwartz *et al.* also observed that jawbone metastases were more likely develop in women (19). However, according to an analysis of 390 cases, male predilection observed in both metastases to the oral mucosa and to the jaws (14). In other studies, there was an equal sex distribution for jawbone metastasis and a male predilection for oral soft tissue (6). In a Korean review, men had a higher rate of metastatic tumors, especially in the jaws (20). The patterns might differ with respect to factors such as race and patient population, or the nature of the research institutions.

A literature review reported that the breasts, lungs, kidneys, and prostate were common sites of primary tumors (13,14,16,17). The present study also showed that the common sources of metastatic carcinomas to the oral region were the lungs and the breasts. According to previous studies, the nature of the primary tumor and the site of metastasis within the oral region and jaws differed between sexes (16,17). In men, the primary tumors were the most commonly found in the lungs and kidneys, and the breast and thyroid tissues were the most common sources of metastasis in women. In the present retrospective analysis, the most common primary site in men was the lungs and in women it was the breast, which was consistent with the previous literatures.

Although the high metastatic rate for the lung and breast is consistent with that of the Western literature, the incidence rate for the liver cancer is inconsistent. Primary liver cancer is more common in Asia than in the West, and it has a poor prognosis with a low chance of survival past 1 year, whether the cancer is primary or secondary (21). We found a higher metastatic rate for the liver as a primary site in both sexes than that reported in the Western literature. McDaniel *et al.* (22) observed only 1 out of the 32 cases of liver cancer metastasis to the oral region. According to the national statistics of Korea, the crude annual incidence rate among the Korean population, the crude mortality rate for liver cancer during 2010 in Korea was very high for both sexes (23). Based on these statistical characteristics of liver cancers in the Korean population, we could speculate on the possible reason behind the high number of oral metastases form the liver. A recent literature review covering the 1983 to 2004 period showed that liver cancer was the source of 27% of total cases in the Korean population, which was even higher than our results (20).

Hepatocellular carcinoma (HCC), the most common primary tumor of the liver, was the most common histologic type of metastatic carcinoma in this study. As a leading cause of death for both the Korean men and women, HCC has a mean survival of much less than 1 year if left untreated (24). To our knowledge, the true incidence of extrahepatic metastases to the jaws and oral region remains unknown. In addition, oral metastasis of hepatocellular carcinoma is uncommon with only 61 publishing cases reported in the literature (25). HCC in advanced stages is very devastating and can easily form metastases to other organs, including the oral region (25). In addition, all HCC cases preferred skeletal bones to soft tissues as their metastatic target in the present study. It is probably due to the preference to the skeletal bones in some metastatic tumors. Of course, when we compared the relative prevalence of primary tumors, the incidence of the primary lesions in that particular population and specific ethnicity should be taken into account.

The jaws, particularly the mandible, were the most frequently involved sites, followed by the oral soft tissues. Consistent with previous findings in a systemic review (18), the present study also revealed that the mandible is the most common location for metastases, with the molar area being the most frequently involved site. The blood vessels in the posterior region of the mandible are well developed, which might help tumor cells to become entrenched. However, the lack of hemopoiesis in the adult maxilla can explain the mandible being much more frequently affected than the maxilla (19). Early lodging of tumor cells occurs in the hemopoietic areas, and tumor cells appear in the most anterior part of the mandibular body or the soft tissues only in advanced stages of the disease (19).

Although there are more published cases of metastases to the jawbone than to the oral soft tissues, metastatic tumors to the oral region might also manifest in the soft tissue (2). In the present analysis, 3 cases were confirmed to involve the oral soft tissue; a tongue, parotid gland, and oropharynx. Of the oral soft tissue tumors, the tongue is one of the most commonly affected sites. Because of richness of blood vessels, the tongue is easier for cancer cells to engraft along the veins (10,26). While the muscle is known to be resistant to metastasis, the tongue is well vascularized and may have the potential to attract metastatic tumor cells (18). We had one case with parotid gland metastases. The major salivary glands reported as relatively common sites for oral metastasis. Among them, parotid gland is the most common salivary gland involved in the metastatic process, followed with a much lower incidence in the submandibular gland (27). According to a literature review of 232 tumors that occurred in the parotid gland, 101 patients had metastatic carcinoma (16). The difference between the two salivary glands can be explained with the absence of lymph nodes in the submandibular gland and the rich lymphatic plexus found within the parotid gland.

In the present study, pain and swelling in the oral region were some of the primary clinical symptoms of the patients. Numerous factors produced by the cancer cells, inflammatory cells, and various kinds of cells derived from bone marrow make it difficult to control the pain (28). In one of the cases that showed metastases to the mandible, which originated from the breast, numbness developed. Numb chin syndrome appears when metastatic tumors are located in the mandible, especially when they invade the mandibular nerve, causing symptoms of numbness and intermittent pain, anesthesia, and hypoesthesia (29). As the metastasis progresses, the patient may feel a sense of severe discomfort in the soft tissues inside the mouth. If these symptoms are observed, we have to consider a metastatic tumor in the differential diagnosis.

Metastatic carcinoma to the oral regions is one of the most devastating diseases, causing significant disfigurement of the patient with severe morbidity and mortality (1,2). In case of metastasis to the oral cavity, the mean survival time was 7 months (13), which is relatively shorter than that for metastasis to other parts of the body. Clinically, the primary origins are often already known to be in an advanced stage when the clinician diagnoses metastatic carcinoma. In the present study, all patients showed that advanced oral cancer with IVC TNM stage. According to several extensive reviews, oral metastasis usually indicates widespread metastases from the primary site (2,6,14). The treatments are also primarily dependent on the site of origin and the degree of metastatic spread (6). If the patient were in advanced malignant disease, a palliative regimen would be necessary to improve the quality of life. When the patient is under the widespread or recurrent metastatic disease, the secondary lesion to the jaws and oral region should be managed conservatively to alleviate the symptoms and preserve oral functional status (30).

## Conclusions

Based on our findings, we can identify the characteristics of metastatic carcinoma in the Korean population. In addition, the clinician and oral pathologist can consider the possible presence of metastases, especially in patients with known malignant tumors. Of course, our estimate of the incidence of oral metastases in the Korean population might not be accurate because the present study was a single-center trial, which could cause some bias in favor of certain primary sites and oral sites. However, it is noteworthy that our research covered a large number of patients during the last 32 years, which was relatively long period. To establish more definitive criteria for diagnosing of carcinoma metastatic to the oral region in the Korean population, well-conducted multicenter, and large population studies are warranted.
